# RETIREES’ MEMORIES OF WORK LIFE AND WELL-BEING AFTER RETIREMENT: A QUALITATIVE INQUIRY

**DOI:** 10.1093/geroni/igz038.1133

**Published:** 2019-11-08

**Authors:** Tina Hahnel, Sabine Hommelhoff, Hsiao-Wen Liao

**Affiliations:** 1 Friedrich-Alexander University, Erlangen-Nürnberg, Germany, Germany; 2 Stanford University, Stanford, California, United States

## Abstract

Reminiscence research has grown immensely in the past 30 years. Yet, research on personal memories of work lives is lacking. This is surprising because work is a crucial aspect of many people’s lives and an important life story chapter (Thomsen, Pillemer, & Ivcevic, 2011). Part of a larger project, the present qualitative study aimed to understand (1) what retirees remember about their work lives and (2) whether and how retirees tie those memories to their current well-being. Six in-depth interviews on lives before and after retirement (4 women and 2 men with different careers, age range 65 to 87 years) were conducted and transcribed verbatim. Findings of a thematic analysis (Braun & Clarke, 2006) revealed that participants reported both big and small stories. They first narrated landmark events (e.g., job loss after the Fall of the German Wall) and continued to recount many little incidents (e.g., a child asking an unretiring teacher if she is now "done with retirement”). Additionally, participants not only reminisced about work itself (i.e., what jobs were like) but equally about workplace relationships (e.g., particularly positive or negative relations with supervisors). Despite difficult times at work, participants reported that they were now at peace with how things went and generally satisfied with their current lives. We discuss how the type (i.e., big or small) and content (i.e., work- or relationship-focused) of retirees’ memories and positive meaning-making (i.e., recounting work lives in a positive light) may contribute to well-being and propose a conceptual model for future research.

